# Supplementary material for: MetaQTL: a package of new computational methods for the meta-analysis of QTL mapping experiments
Source: BMC Bioinformatics. 2007 Feb 8;8:49. doi: 10.1186/1471-2105-8-49 (PMC1808479; doi:10.1186/1471-2105-8-49)
Supplement: Additional File 7 — MetaQTL Package : jar file and tutorial. This Zip archive contains both the MetaQTL JAR file and the files of the tutorial. [file 1471-2105-8-49-S7.zip › org.inra.metaqtl/doc/org/metaqtl/graph/QtlLayerBuffer.html]

QtlLayerBuffer


|  |  |  |
| --- | --- | --- |
| **Overview** \| **Package** \| **Class** \| **Use** \| **Tree** \| **Deprecated** \| **Index** \| **Help** | |  |
| **PREV CLASS**   **NEXT CLASS** | **FRAMES**    **NO FRAMES**     **All Classes** |  |
| SUMMARY: NESTED \| FIELD \| CONSTR \| METHOD | DETAIL: FIELD \| CONSTR \| METHOD |  |


---


## org.metaqtl.graph Class QtlLayerBuffer

```
java.lang.Object
  org.metaqtl.graph.QtlLayerBuffer
```

---

``` public class QtlLayerBuffer extends java.lang.Object ```

---

**Constructor Summary**

`QtlLayerBuffer()`


| **Method Summary** | |
| --- | --- |
| `boolean` | `addQtl(java.awt.Graphics2D graph, QtlUnit unit)`             Try to add a qtl unit to the configuration. |
| `boolean` | `canAddMoreQtl(double ymax)`             Retruns true if more Qtl can be added to the configuration, false otherwise. |
| `QtlLayer` | `getLayer(double x, double y)`             Returns a QtlLayer which represent the contents of the buffer. |

**Methods inherited from class java.lang.Object**

`clone, equals, finalize, getClass, hashCode, notify, notifyAll, toString, wait, wait, wait`

**Constructor Detail**

### QtlLayerBuffer

```
public QtlLayerBuffer()
```


**Method Detail**

### addQtl

```
public boolean addQtl(java.awt.Graphics2D graph,
                      QtlUnit unit)
```

:   Try to add a qtl unit to the configuration.
    If the qtl has been adde then the method returns true
    otherwise it returns false.

    :   **Parameters:**: `graph` -: `font` -: `unit` - **Returns:**

---


### canAddMoreQtl

```
public boolean canAddMoreQtl(double ymax)
```

:   Retruns true if more Qtl can be added to the configuration,
    false otherwise.

    :   **Returns:**

---


### getLayer

```
public QtlLayer getLayer(double x,
                         double y)
```

:   Returns a QtlLayer which represent the contents of
    the buffer.

    :   **Returns:**


---


|  |  |  |
| --- | --- | --- |
| **Overview** \| **Package** \| **Class** \| **Use** \| **Tree** \| **Deprecated** \| **Index** \| **Help** | |  |
| **PREV CLASS**   **NEXT CLASS** | **FRAMES**    **NO FRAMES**     **All Classes** |  |
| SUMMARY: NESTED \| FIELD \| CONSTR \| METHOD | DETAIL: FIELD \| CONSTR \| METHOD |  |


---
